# The first thousand days in a newly established radiation oncology clinic: an evaluation study

**DOI:** 10.3332/ecancer.2025.1875

**Published:** 2025-03-18

**Authors:** Harun Demir, İbrahim Babalıoğlu, Aslı Şahbaz, Mehmet Koç

**Affiliations:** 1 Department of Radiation Oncology, Konya City Hospital, Konya 42000, Turkey; 2 Department of Radiation Oncology, Meram Faculty of Medicine and Konya Provincial Health Directorate, Necmettin Erbakan University, Konya 42005, Turkey

**Keywords:** cancer, epidemiology, radiation oncology

## Abstract

**Aim:**

The study aimed to determine the distribution of cancer and treatment characteristics in a recently started radiation oncology (RO) clinic and to examine the differences according to age groups and gender.

**Method:**

The medical records of 2,434 patients who underwent treatment at Konya City Hospital’s RO clinic between June 2021 and March 2024 were retrospectively examined. Descriptive analyses and the chi-square method were applied.

**Results:**

The median age was 63 years. Age groupings revealed that, in young adults, breast cancer and central nervous system malignancies were the most common (56.1, 14.6%), in adults, breast cancer and lung cancer (42.0, 24.0%), and in older adults, lung cancer and gastrointestinal system (GIS) cancers (33.6, 17.4%) (*p* = 0.00). The metastatic stage rate was higher in men at the time of initial diagnosis (*p* = 0.00). Lung cancer was more common in metastatic patients, whereas breast cancer was more common in non-metastatic patients (*p* = 0.00). Neoadjuvant RT was most often used in GIS malignancies (*p* = 0.00), adjuvant RT was most often used in breast cancer, and definitive, palliative RT was most often used in lung cancer. The first stage of the disease had not been associated with the distance to the treatment facility (*p* = 0.43).

**Conclusion:**

Our research has revealed significant differences in the distribution of cancer, stage and the role of radiotherapy according to both age groups and gender in the practice of RO. These findings can be utilised as a model for more efficient health strategising.

## Introduction

The societal significance of diseases is determined by their incidence, cost, morbidity and mortality [1]. In this context, cancer today appears as a global health problem that affects many individuals, including those in developed societies. The increase in life expectancy, which is a result of improved quality of life and advancements in healthcare, leads to a higher prevalence of various diseases, including cancer. Worldwide, between 19 and 20 million individuals receive cancer diagnoses each year, and approximately 10 million people die from the disease [2].

The frequency and type distribution of cancer vary throughout societies. The International Agency for Research on Cancer’s data indicates that Turkey saw more than 240 thousand new cancer cases in 2022. The three most common kinds of cancer are breast, lung and prostate [3]. In the 2018 data of our country’s Ministry of Health, Cancer Control Department, the age-standardised cancer rate is reported as 262.4 per 100,000 in men and 188.0 per 100,000 in women. The data indicates that the prevalence of cancer in our country is comparatively lower among both males and females when compared to regions such as Europe, Canada, America and Australia [4].

Many studies indicate that there are significant differences in cancer incidence between regions in our country as well as globally. In a study investigating the epidemiology of lung cancer in our country, for instance, Izmir had the greatest incidence of men and Diyarbakır the lowest; Sivas and Diyarbakır had the highest and lowest instances, respectively, of women. The situation is associated with a number of factors, including the socioeconomic structure, the fact that the Izmir region experiences regular immigration and the extensive tobacco production there [5].

A multidisciplinary approach involving many fields, especially surgery, medical oncology and radiation oncology (RO), is essential in cancer treatment. RO is involved in several stages of the treatment process, such as definitive treatments for curative purposes, pre- or post-operative adjuvant treatments and palliative treatments for advanced-stage patients. RO is one of the fields most affected by technological developments due to the modern linear accelerator devices used.

**Figure 1.**
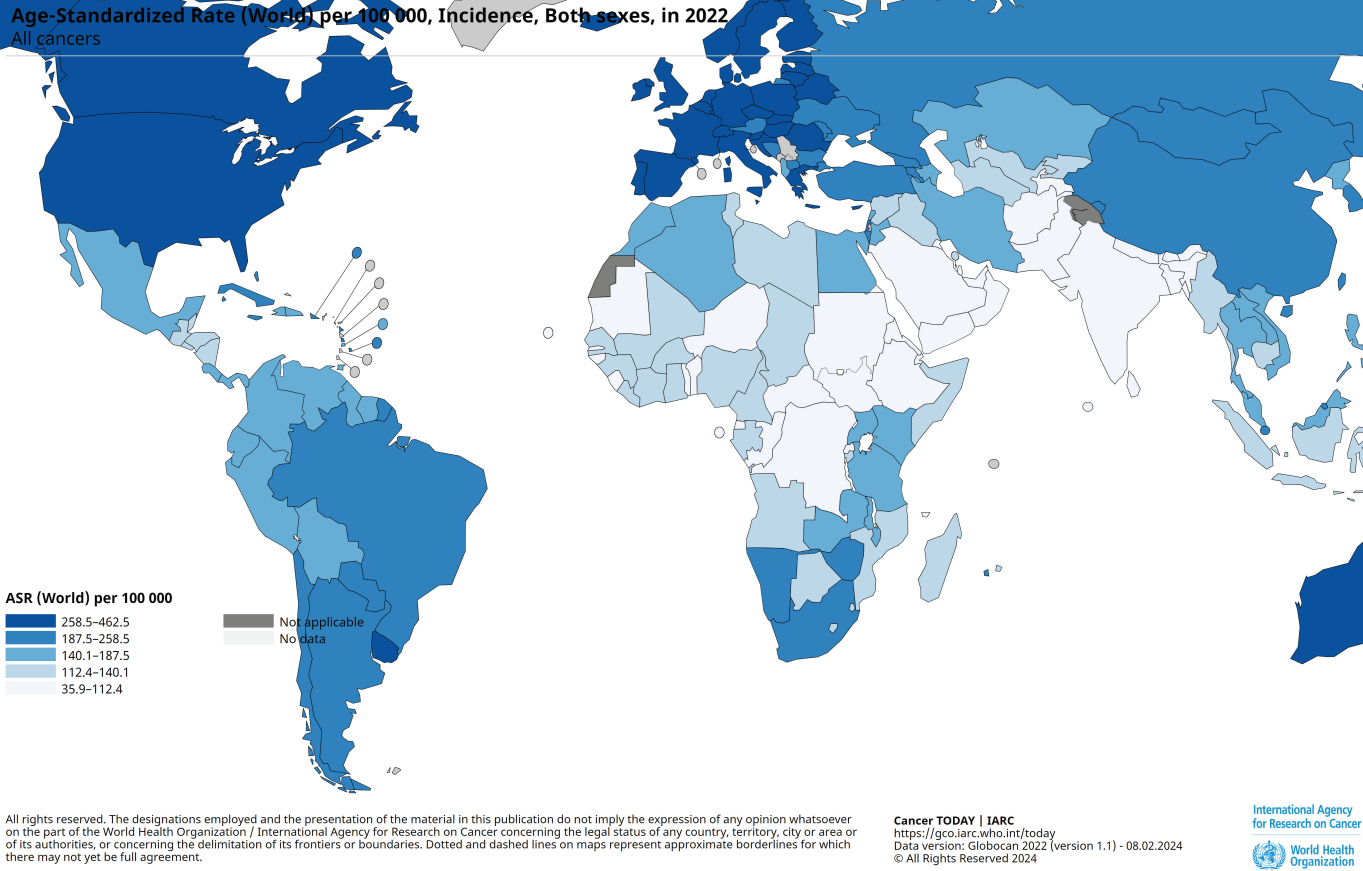
Distribution of radiotherapy centers in Turkey (According to 2011 data) [6]. *Numbers indicated on each region refers to million population in the region. **Red star, indicates Konya region.

Goksel *et al* [6] published a study on the current status and future perspectives of RO facilities in Turkey in 2011. Accordingly, considering different socioeconomic factors, population size and patients’ travel-related behavior, the country was divided into 29 medical regions by the Ministry of Health. Figure 1 illustrates the situation at that time. Our center is located in the Konya region, and radiotherapy was administered using the Varian DHX device (Varian Medical Systems, Inc., Palo Alto, CA, USA) in the previous clinic from 2010 to 2020. Only 3D conformal treatments and intensity modulated radiation therapy (IMRT) treatments could be performed here. Three-dimensional pre-treatment image verification could only be done with 2-dimensional images. Stereotactic radiotherapy could not be performed due to reasons such as the lack of advanced respiratory motion management and inadequacies in the planning system. Similarly, considering the inadequacy of the existing hospital’s physical facilities against the increasing number of patients, it was decided by the administrative authorities to establish a new hospital and a new RO clinic.

Konya City Hospital was established in October 2020 and provides healthcare services to a population of approximately three million, including the surrounding provinces. The RO clinic has commenced admitting patients in June 2021. The Elekta Versa HD linear accelerator (LINAC) with 6, 10 and 15 MV filtered photons; 6 and 10 MV unfiltered (FFF) photon energies; and 6, 8, 10, 12 and 15 MeV electron energies were installed in the new clinic. The new LINAC can achieve high dose rates by using filter-free photons, and with its suitable multileaf collimator (MLC) structure, three- and four-dimensional cone beam computed tomography (CT) portal imaging system and hexapod six-dimensional table operating with sub-millimeter precision, many treatments can be applied, especially volumetric modulated arc therapy and stereotactic radiotherapy (RT). In addition, the new modern hospital, which meets current needs, has provided the opportunity to give much more modern treatments to more patients with shorter treatment times.

As a representative sample, this study allows the frequency of cancer and the more common cancer types in our region to be known in terms of RO. In this way, it is aimed at contributing to the planning of health services, such as researching the factors that play a role in etiology, cancer prevention, early diagnosis and the determination of treatment approaches.

## Materials and methods

This study analysed the demographic features of patients who received treatment at our clinic from June 2021 to March 2024. A total of 2,434 patients’ medical records were retrospectively analysed. The data of 714 patients (29.4%) who did not receive radiotherapy in our clinic due to reasons such as lack of radiotherapy indication and application to a different center were not included in the analysis. The diagnosis, stage, age, gender, place of residence and treatment characteristics of the patients were assessed. Statistical techniques, descriptive analyses and the chi-square method were used. A significance level of *p* < 0.05 was selected to determine statistical significance. The data were analysed with SPSS 22.0 statistical software.

Ethics committee approval was received for the study from the Necmettin Erbakan University Faculty of Medicine Non-Drug and Non-Medical Device Research Ethics Committee dated 17.05.2024 and numbered 2024/4968. This research complies with institutional policies, Research and Publication Ethics and the principles of the Declaration of Helsinki.

## Results

The females comprised 50.8% of the total number of patients (*n* = 874), and the female-to-male ratio is 1.01. All patients had median ages of 63 years (range 9–94 years), 57 years (range 16–94 years) for women and 67 years (range 9–91 years) for men.

Patients were examined in three groups according to their age: young adult (≤40), adult (41–64) and advanced age (≥65). The largest group is adults (49.0% *n*:843), followed by elderly patients (43.8% *n*:754) and young adults (7.2% *n*:123). A significant difference was detected between age groups and gender. Accordingly, female gender was more prevalent among young adults (79.7%) and adults (59.8%), while the majority of older patients were males (63.9%) (*p* = 0.00).

**Figure 2.**
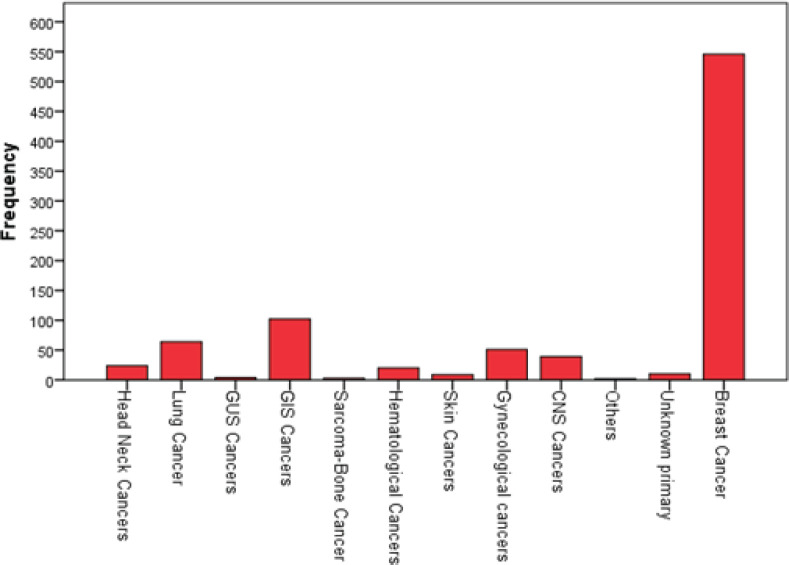
Distribution of cancer types in women.

**Figure 3.**
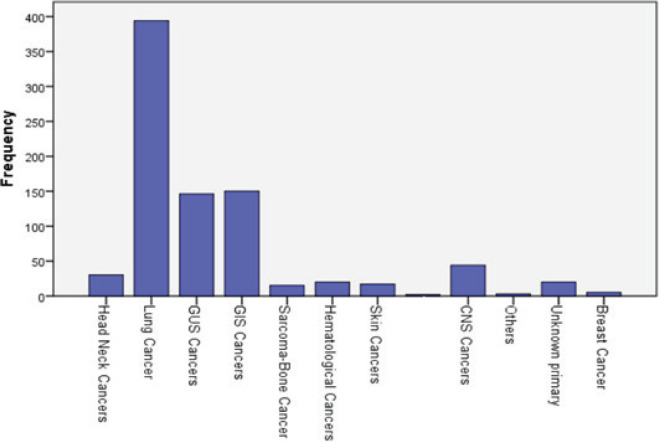
Distribution of cancer types in men.

The most prevalent cancers, according to frequency, were breast cancer (32%), lung cancer (26.6%), gastrointestinal system (GIS) cancers (14.7%), genitourinary system (GUS) cancers (8.7%) and central nervous system (CNS) malignancies (4.8%). The three most common cancers in men were lung (46.6%), GIS (17.7%) and GUS (17.3%), while in women they were breast (62.5%), GIS (11.7%) and lung (7.3%) (Figures 2 and 3).

When the cancer types were evaluated in detail, nasopharynx, larynx and lip cancers were the most prevalent head and neck cancers (29.6%, 18.5% and 14.8%), respectively, while definitive (50%) and adjuvant (33.3%) radiotherapy were the most often used treatments. For the thorax region, lung cancer was the most common (98.5%) and patients were mostly treated with palliative radiotherapy (51.6%) and definitive radiotherapy (34.1%). Breast cancer constituted 32% of all patients, and adjuvant radiotherapy (86.8%) and palliative RT (12.2%) were most frequently performed.

The most common malignancies in the GIS were pancreatic, rectal and stomach cancers (50.4%, 17.5% and 9.9%), respectively, while the most often used treatments were neoadjuvant (33.7%) and palliative (27.1%) radiotherapy. Prostate, bladder and kidney cancer were the most common GUS malignancies (78.0, 12.7 and 8.0%, respectively), and patients were treated with definitive (49.3%) and palliative (36.7%) radiation the most often. Cervix and endometrium (49.1% and 41.5%) were the most common gynecological malignancies; patients were most often treated with adjuvant (47.2%) and definitive radiation (30.2%).

Age group-based diagnosis distribution showed a statistically significant difference. Young adults had the most breast, CNS and hematological malignancies (56.1%, 14.6% and 6.5%); people had the most breast, lung and GIS cancers (42.0%, 24.0% and 13.2%); and older adults had the most lung, GIS and breast cancer (33.6%, 17.4% and 17.0%). (*p* = 0.00) (Table 1). Furthermore, the disease’s initial stage differed significantly by age group. Accordingly, most metastatic patients were older adults (54.7% *n* = 321), and most non-metastatic patients were adults (52.4% *n* = 594) (*p* = 0.00).

The patients were categorised into two categories, metastatic and non-metastatic, according to the initial stage of cancer. At the beginning, 34.1% (*n* = 587) were in the metastatic stage; of them, 32.5% were women (*n* = 191) and 67.5% were men (*n* = 396). According to gender, men’s metastatic stage rate was 46.8%, and women’s was 21.9%, a statistically significant difference (*p* = 0.00). A significant difference was also observed in the first stages based on the types of cancer. Accordingly, lung cancer accounted for the majority of metastatic patients (45.8%, *n* = 269), whereas breast cancer comprised the majority of non-metastatic patients (42.4%, *n* = 478) (*p* = 0.00).

According to the modality of radiotherapy, the patients were divided into five groups. Adjuvant radiotherapy was given to 41.5% of patients, palliative to 30.3%, definitive to 19.2%, neoadjuvant to 4.9% and stereotaxic to 4.2%. Radiotherapy modality distribution differed significantly by cancer type. The use rates for definitive, palliative and stereotaxic radiotherapy were highest for lung cancer (47.3%, 45.4% and 50.8%, respectively), followed by adjuvant radiotherapy for breast cancer (67.0%) and neoadjuvant radiotherapy for GIS cancers (100%) (*p* = 0.00). Based on the frequency of radiotherapy treatments administered per patient, it was determined that those who underwent three or more RTs (mostly palliative) were most commonly with lung cancer (47.4%) and breast cancer (18.0%), respectively (*p* = 0.00) (Table 2).

The patients were evaluated in four groups according to the distance of their settlements to our center. Accordingly, 57.7% of the patients applied from the central region (≤50 km), 14.2% from nearby districts (50–100 km), 21.6% from distant districts (100–200 km) and 6.4% from outside the city (>200 km). In the evaluation of location and disease in the initial stage, the metastatic stage rates were 33.2%, 33.5%, 40.9% and 34.1% from the central region to the city outside, with no significant difference (*p* = 0.43).

## Discussion

Cancer is one of the most significant public health issues in modern times, characterised by its frequent incidence and high rates of mortality and morbidity. According to the World Health Organisation (WHO) report, cancer ranks as the second leading cause of mortality globally and within our country. It is responsible for the deaths of over 10 million individuals, as per the data from 2020 [7].

The incidence rate of cancer types varies widely between societies depending on geographical location, race, gender and age. A comprehensive understanding of cancer statistics and epidemiology is critical for optimising the utilisation of available resources all over the entire cancer fight, including screening, diagnosis, treatment and rehabilitation. In this context, many national and international reports are published, and these reports are updated periodically [4, 8]. According to the data of the Cancer Control Department of the Turkish Ministry of Health, the cancer incidence in our country is around 1/2 or even 1/3 of the incidence in developed Western countries. This difference in incidence is associated with the higher proportion of young people in the age distribution of our country’s population. However, along with the decreasing population growth rate, there is a tendency for population aging and increasing cancer incidence in Turkey [9].

According to the restructuring program data of Turkish oncology service units, approximately 55%–60% of patients newly diagnosed with cancer receive radiotherapy at least once during the entire treatment period. Palliative radiotherapy is administered in 20%–25% of these cases following the initial series of radiotherapy, typically due to the presence of brain and bone metastases [10]. The WHO and the International Atomic Energy Agency initiated a baseline country survey on medical devices in 2010 to assess high-cost medical devices in Member States, including radiotherapy equipment like linear accelerators and Cobalt-60 [11]. According to data from that time, the number of treatment devices in Turkey is estimated to be approximately 2 devices per 1 million population. In this context, a restructuring program was implemented to meet the need for radiotherapy devices in a projection covering the years 2010–2023 [10]. In this study, the data of patients who underwent radiotherapy at our center, which was established in 2021, in a period exceeding three years was analysed. It evaluates age and gender demographics, stage distribution of cancer types and the distribution of treatment modalities by cancer type and age.

There was a balanced distribution in terms of gender in the patients (female-male ratio 1.01), and the median age was 63 (9–94) years. However, the number of older patients is more than six times that of young adults. These data support the hypothesis that aging is a fundamental factor in cancer development, especially due to the increasing accumulation of risks [12]. As the rate of life expectancy rises and individuals live longer, cancer emerges as a significant public health concern among the elderly. Given this situation, it is crucial to take into account the potential burden this may have on society when making future health plans.

According to 2023 global cancer statistics, the cancer types with the highest incidence are breast cancer (11.7%), lung cancer (11.4%) and prostate cancer (7.3%) [2]. In our study, the four most common cancers were breast cancer (32%), lung cancer (26.6%), rectal cancer (7.4%) and prostate cancer (6.8%). This difference shows that in RO practice, rectal cancer cases are more common than prostate cancer in our clinic. Again, the fact that early-stage laryngeal cancer cases in our institution mainly receive surgical treatments shows that the lower rate of larynx cancer (18.5%) in head and neck cancer patients compared to nasopharynx cancer (29.6%) is explained.

According to global gender-based statistics, the most common cancers in men are lung (14.3%), prostate (14.1%) and non-melanoma skin cancers (7.2%), and in women, the most common cancers are breast (24.5%), lung (8.4%) and cervix cancer (6.5%) [2]. In our study, the three most common malignancies among women are lung cancer, breast cancer and gastrointestinal (GI) cancers. By contrast, the top three most common malignancies among men are lung cancer, GIS tumours and GUS cancers.

The cancer statistics report from the Ministry of Health in Turkey reports that among individuals aged 15–24, testicular cancer and Hodgkin lymphoma are the predominant types of cancer in men, while thyroid cancer and Hodgkin lymphoma are the most prevalent in women. The most common cancers in 24- to 49-year-olds are colorectal, testicular and thyroid in men and breast and thyroid in women. Between 50 and 69, men develop mostly prostate and lung cancers, while women develop colorectal and breast cancers [4]. According to this study, which represents the RO routine, breast cancer and CNS malignancies are the most common in young adults, breast cancer and lung cancer are the most common in the adult group and lung cancer and GI cancer are the most common in the elderly. It is attributed to the fact that these differences are mainly related to the effectiveness of radiotherapy in the treatment of the relevant cancer type and regional incidence variations.

There are significant differences in the clinical course between cancer types. Although some types remain asymptomatic for a long time, others show an aggressive course and metastasise earlier. In a recently published multicenter study, the relationship between cancer types and stage of diagnosis was examined. This study reports that breast, lung and colorectal cancers are diagnosed at a more advanced stage in China compared to the USA. Additionally, the rate of advanced-stage patients was found to be higher in men than in women [13]. In our study, the metastatic stage rate of men at initial diagnosis was found to be significantly higher than that of women (46.8% versus 21.9%, *p* = 0.00). Furthermore, a significant difference was found between cancer types and the first stage, with lung cancer cases presenting mostly at the metastatic stage (59.0%) and breast cancer cases mostly presenting at the non-metastatic stage (86.8%).

Many studies have been conducted investigating the effects of differences in access to health services between rural and urban regions on the treatment outcomes of cancer patients [14]. There are also studies reporting that cancer death rates are higher in rural areas than in urban areas [15]. Upon analysing the data from our study, it is obvious that almost one-third of the patients live in places located 100 km or more away from our center. Although there is no significant difference between the location of residence and the first stage in our analyses, it is imperative to conduct multicenter studies with extensive involvement to mitigate disparities in healthcare access.

A multidisciplinary approach is crucial in the treatment of cancer. In this context, surgical oncology, medical oncology and RO constitute the three most important pillars, along with many other branches. RO is the main branch of science that investigates the effects of ionising radiation on cancer and aims to treat cancer. Although RO is widely used in the treatment of many types of cancer, there is no research in the literature that especially analyses patient statistics in this field and the distribution of treatment types. In this respect, our study completes an important deficiency in that it examines in detail the demographic data, disease and treatment characteristics of all patients who received treatment in the first thousand days of our center, as a public hospital that is accessible to all segments of society.

## Conclusion

Consequently, cancer, which is one of the most important health problems globally today, creates a major problem for healthcare systems with both financial and workforce burdens. In this sense, it is important for all societies, including developed countries, to use existing resources in the most effective way. To effectively fight cancer, one must know the regional and national prevalence of the disease and the most significant diagnosis, treatment and care areas, that is, knowing cancer statistics. In this respect, this study contains significant regional data in a specialist field that offers expensive treatments, such as RO. Strengthening the data obtained with multicenter studies, including centers from different regions, will enable both the improvement of health service quality and the efficient utilisation of resources.

## Conflicts of interest

None.

## Funding

None.

## Figures and Tables

**Table 1.** Distribution of cancer types based on age groups in all patients.

	Age groups		
Type	≤40	41–64	≥65
	Percentage (*n*)	Percentage (*n*)	Percentage (*n*)
Head neck cancers	4.1 (*n*:5)	3.1 (*n*:26)	3.1 (*n*:23)
Lung cancer	2.4 (*n*:3)	24.0 (*n*:202)	33.6 (*n*:253)
Breast cancer	56.1 (*n*:69)	42.0 (*n*:354)	17.0 (*n*:128)
GUS cancers	2.4 (*n*:3)	4.0 (*n*:34)	15.0 (*n*:113)
GIS cancers	8.1 (*n*:10)	13.2 (*n*:111)	17.4 (*n*:131)
Sarcoma-bone cancer	3.3 (*n*:4)	0.6 (*n*:5)	1.2 (*n*:9)
Hematological cancers	6.5 (*n*:8)	2.0 (*n*:17)	2.0 (*n*:15)
Skin cancers	0.0 (*n*:0)	0.9 (*n*:8)	2.4 (*n*:18)
Gynecological cancers	1.6 (*n*:2)	3.6 (*n*:30)	2.8 (*n*:21)
CNS cancers	14.6 (*n*:18)	4.3 (*n*:36)	3.8 (*n*:29)
Cancer of unknown primary	0.8 (*n*:1)	2.3 (*n*:20)	1.9 (*n*:14)
Sum	100 (*n*:123)	100 (*n*:843)	754 (*n*:35)

GUS, genitourinary system; GIS, gastrointestinal system; CNS, central nervous system

**Table 2.** Distribution of the radiotherapy based on age groups in all patients.

	Age groups		
Modality of radiotherapy	≤40	41-64	≥65
	Percentage (*n*)	Percentage (*n*)	Percentage (*n*)
Definitive	13.8 (*n*:17)	14.7 (*n*:124)	25.1 (*n*:189)
Adjuvant	69.9 (*n*:86)	51.2 (*n*:432)	25.9 (*n*:195)
Neoadjuvant	2.4 (*n*:3)	4.3 (*n*:36)	6.1 (*n*:46)
Palliative	10.6 (*n*:13)	26.7 (*n*:225)	38.3 (*n*:289)
Stereotaxic	3.3 (*n*:4)	3.1 (*n*:26)	4.6 (*n*:35)
Sum	100 (*n*:123)	100 (*n*:843)	754 (*n*:35)
